# Hinge length contributes to the phagocytic activity of HIV-specific IgG1 and IgG3 antibodies

**DOI:** 10.1371/journal.ppat.1008083

**Published:** 2020-02-24

**Authors:** Thach H. Chu, Andrew R. Crowley, Iara Backes, Cheryl Chang, Matthew Tay, Thomas Broge, Marina Tuyishime, Guido Ferrari, Michael S. Seaman, Simone I. Richardson, Georgia D. Tomaras, Galit Alter, David Leib, Margaret E. Ackerman

**Affiliations:** 1 Thayer School of Engineering, Dartmouth College, Hanover, New Hampshire, United States of America; 2 The Geisel School of Medicine at Dartmouth, Lebanon, New Hampshire, United States of America; 3 Duke Human Vaccine Institute, Duke University Medical Center, Durham, North Carolina, United States of America; 4 Ragon Institute of MGH, MIT, and Harvard, Cambridge, Massachusetts, United States of America; 5 Beth Israel Deaconess Medical Center, Boston, Massachusetts, United States of America; 6 Centre for HIV and STIs, National Institute for Communicable Diseases, Johannesburg, Gauteng, South Africa; King's College London, UNITED KINGDOM

## Abstract

Antibody functions such as neutralization require recognition of antigen by the Fab region, while effector functions are additionally mediated by interactions of the Fc region with soluble factors and cellular receptors. The efficacy of individual antibodies varies based on Fab domain characteristics, such as affinity for antigen and epitope-specificity, and on Fc domain characteristics that include isotype, subclass, and glycosylation profile. Here, a series of HIV-specific antibody subclass and hinge variants were constructed and tested to define those properties associated with differential effector function. In the context of the broadly neutralizing CD4 binding site-specific antibody VRC01 and the variable loop (V3) binding antibody 447-52D, hinge truncation and extension had a considerable impact on the magnitude of phagocytic activity of both IgG1 and IgG3 subclasses. The improvement in phagocytic potency of antibodies with extended hinges could not be attributed to changes in either intrinsic antigen or antibody receptor affinity. This effect was specific to phagocytosis and was generalizable to different phagocytes, at different effector cell to target ratios, for target particles of different size and composition, and occurred across a range of antibody concentrations. Antibody dependent cellular cytotoxicity and neutralization were generally independent of hinge length, and complement deposition displayed variable local optima. *In vivo* stability testing showed that IgG molecules with altered hinges can exhibit similar biodistribution and pharmacokinetic profiles as IgG1. Overall, these results suggest that when high phagocytic activity is desirable, therapeutic antibodies may benefit from being formatted as human IgG3 or engineered IgG1 forms with elongated hinges.

## Author summary

Target binding and receptor binding domains of antibodies are separated by a hinge region that differs among IgG subclasses. Among these, IgG3 has a uniquely elongated hinge, whose role in an immunoglobulin’s ability to facilitate different immune functions is incompletely understood. We evaluated the effect of varied hinge length and composition on diverse antibody activities and found that extended hinges enhance phagocytosis, a process through which the immune system can clear potentially harmful particles such as viruses. This enhancement was observed across diverse effector cell types, target particles, and for multiple HIV-specific antibodies. Antibodies with extended hinges showed *in vivo* persistence and localization in a mouse model similar to those with natural hinges. Consequently, immune responses or antibody therapies expected to benefit from a strong phagocytic response, such as viral sequestration, may be enhanced naturally by the IgG3 subclass or artificially by hinge extension. This work expands knowledge of the role that IgG3 plays in immunity and provides a framework for hinge engineering to improve antibodies.

## Introduction

Immunoglobulin G (IgG) proteins, which can mediate neutralization, complement-dependent cytotoxicity (CDC), antibody-dependent cellular cytotoxicity (ADCC), and antibody-dependent cellular phagocytosis (ADCP), among other activities, are key components of the adaptive immune system. This diversity of activities is mirrored by diversity in structure and sequence. Structurally, IgG antibodies are comprised of a pair of variably disulfide bond-bridged heavy and light chains that combine to form two binding sites for their cognate antigen, called fragment antigen binding (Fab) domains. Those domains are linked to a single, structurally asymmetric fragment crystallizable (Fc) domain that is responsible for interactions with innate immune, recycling, and transport receptors. While Fab sequences present essentially infinite sequence diversity, only four major Fc domain subtypes, or subclasses, are expressed in human IgG molecules.

Collectively, the combination of Fab and Fc properties of an antibody (Ab) define its *in vitro* and *in vivo* activity profile. Neutralization is driven by Fab binding that results in inhibition of key interactions [1–5], and can render pathogens non-infectious. Requiring only the interaction between an antibody and a target antigen makes neutralization fundamentally distinct from effector functions, which rely on recruitment of either additional soluble components or cellular factors and receptors. Complement–dependent antibody activities, which can ultimately result in cytolysis based on the formation of pores in the membrane of an antibody-targeted pathogen, can be initiated by interactions between the Fc domain and soluble complement factors in serum. This activity can be further enhanced by Fc-Fc driven multimerization interactions [6, 7] that may be favored as a result of the proximal binding of multiple antibody molecules to multiple antigen molecules arrayed on the surface of an infectious particle. Cellular effector functions such as ADCP and ADCC are similarly driven by proximal ligation of multiple antibodies with Fc receptors expressed on a wide range of innate immune cell types. Human Fcɣ receptors (FcɣR) are comprised of high and low affinity, activating and inhibitory family members that elicit a number of anti-pathogen activities from phagocytes, neutrophils, and NK cells, among others upon binding [8–11].

The binding affinities of Fab and Fc domains for antigen and FcɣR, respectively, both of which evolve over time in a maturing immune response, are known to be key variables in defining and regulating antibody activity. Though modified by Fc domain glycosylation at a conserved motif, the effector functions of the limited number of human IgG subclasses are generally predictable, with IgG1 and IgG3 exhibiting higher affinity for FcɣR and consequently greater effector function than their IgG2 and IgG4 counterparts. Moving beyond these useful approximations, however, a number of recent studies aimed at defining the activity of subclass-switched HIV-specific antibodies have observed enhanced phagocytic activity of human IgG3 relative to IgG1 [12–14].

This enhanced activity is particularly intriguing as it points to potential clinical relevance of phagocytosis given the association of IgG3 responses with decreased risk of infection among RV144 HIV vaccine recipients [15], and associations between IgG3 responses and polyfunctional antibodies in HIV vaccine recipients [16] and infected subjects [17]. Associations between IgG3 responses and improved infectious disease outcomes have also been observed for chikungunya and plasmodium infection [15, 18–21], among others. Furthermore, individuals with reduced or deficient serum levels of IgG3 tend to have recurrent upper respiratory infections [22–25]. Collectively, these clinical associations and subclass switch experiments suggest the functional importance of the IgG3 subclass.

Interestingly, while many species express IgG subtypes with different effector function profiles, the hinge region between the Fab and Fc of the human IgG3 molecule is structurally unique among human and non-human immunoglobulin isotypes and subclasses [26–28]. The most common IgG3 hinge allotype in humans is encoded by four exons; the first exon being a 17 amino acid long region followed by a triplicate repeats of a 15 amino acid exon for a total of 62 amino acids [29, 30]. Relative to other subclasses, this sequence extension also results in a physical extension between the Fab and Fc regions that can be observed through electron microscopy [31] and approximately doubles the length of the molecule [32]. While the presence of prolines and cysteines generates rigidity within the local amino acid structure, the overall IgG3 hinge is quite flexible. As a result, interdomain position angles between Fab-Fab and Fab-Fc domains sample a wider range for IgG3 than any other subclass [33].

Given this unique structural diversity, a number of studies have varied hinge composition with an emphasis on improving antibody functions. The disulfide bond configuration of the IgG2 hinge has been associated with imparting activity changes, for example, agonistic capacity [34]. Previous work on the IgG3 hinge has demonstrated that while the hinge is not required for efficient phagocytic activity [35], its composition can affect multiple effector functions [35–42]. IgG3 has been shown to possess enhanced complement-mediated lysis relative to IgG1 specifically in cases of sparse antigen density [43, 44]. However, truncation of the IgG3 hinge has also been shown to result in enhanced complement deposition [39, 40]. For other functions, proximal or distal positioning with respect to the target cell membrane has been found to be of some importance. For example, ADCC activity has been observed to be potentiated by membrane proximal and phagocytosis improved for membrane distal epitopes [45].

Despite such structural and functional distinctions, IgG3 is the only human gamma globulin that has not been advanced clinically as a monoclonal. There are likely several reasons for this notable absence, including concerns about half-life, proteolytic susceptibility of the hinge and allotypic polymorphisms [46–49]. Chief among these perhaps, the majority of IgG3 allotypes bear an Fc sequence in which a histidine residue key to pH-dependent binding to the neonatal Fc receptor (FcRn) responsible for antibody recycling and the long half-life of IgGs [50, 51] is substituted with an arginine. Thus, in early studies, rapid plasma clearance was presumed to be a global attribute of IgG3. This position also impacts recognition by protein A, the receptor used for affinity chromatography in antibody purification, resulting in both a clinical and a technical basis to disfavor the development of IgG3 therapeutics. Now that this phenotype has been attributed to a single amino acid polymorphism and natural IgG3 allotypes have been defined that exhibit long, IgG1-like half-lives [51], there is renewed interest in the clinical prospects of the IgG3 subclass [52–55].

Here we seek to complement and extend subclass switching studies and the previous work that has evaluated variation specifically within the IgG3 hinge [35–41]. To more completely study the contribution of the IgG3 hinge to antibody effector function, HIV-specific IgG3 antibodies with natural and non-natural hinge lengths were generated, swapped into IgG1 backbones and different Fab contexts, and assayed for their activity across diverse effector functions. We demonstrate that phagocytic activity correlates directly with the number of core hinge repeats, while other functions such as neutralization, ADCC, and complement deposition are not globally improved by increasing hinge length. Lastly, we explore the potential translational suitability of hinge length modification by determining the impact of addition of core repeats on antibody stability *in vitro* and *in vivo*.

## Results

### Antibody subclass is an important factor in ADCP potency

Antibodies mediate a variety of effector functions that have the potential to contribute to protection from infection. While IgG1 and IgG3 are the classical effector and complement-activating IgG subclasses, previous studies have found that IgG3 tends to have increased ADCP activity as compared to IgG1 [12, 13, 56]. To evaluate the generalizability of this observation, IgG1 and IgG3 forms of two different HIV envelope glycoprotein-specific antibodies were generated; VRC01 [57], a broadly neutralizing antibody that binds to and blocks the CD4 binding site on the HIV-1 envelope glycoprotein, and 447-52D [58, 59], a more moderately neutralizing antibody specific to the envelope protein’s V3 variable loop. These antibodies were assayed for their ADCP potency in a widely utilized assay of phagocytosis [60] that assesses the antibody-dependent uptake of antigen-functionalized beads by the monocytic THP-1 cell line [61]. Despite reliance on recombinant antigen, likely at high density, and fluorescent beads of an intermediate size between virions and infected cells (among other factors that likely poorly mimic relevant HIV-1 targets *in vivo*), this reproducible assay has demonstrated an association with protection in a number of non-human primate (NHP) vaccine studies [62–64] and in HVTN505 [65], suggesting that it may nonetheless serve as a biologically relevant means to probe this effector function.

**Fig 1:**
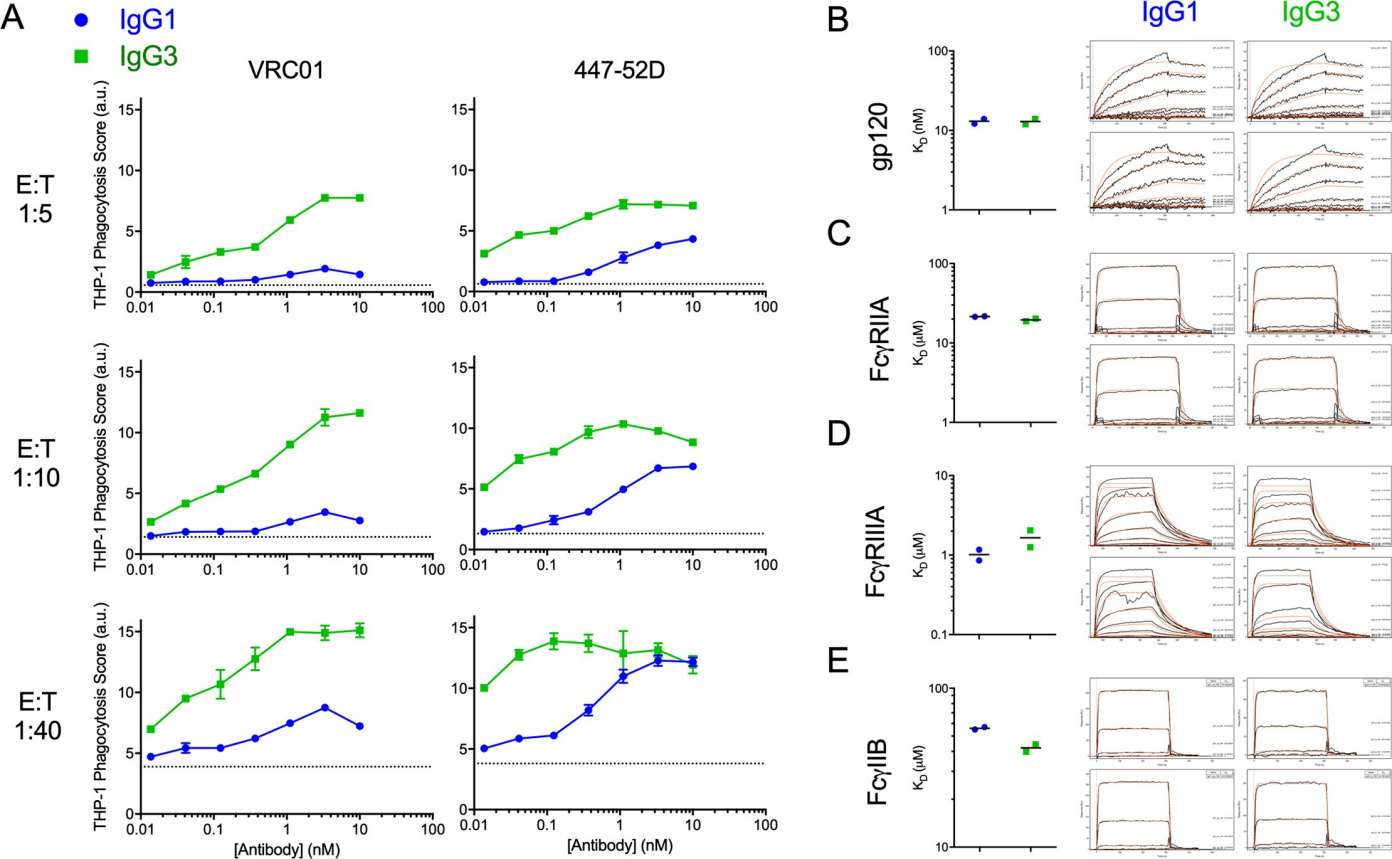
IgG3 Abs exhibit enhanced ADCP despite similar antigen and FcɣR binding affinities. **A.** Phagocytic activity of subclass-switched forms of VRC01 (left) and 447-52D (right) antibodies over a range of effector to target (E:T) ratios in the THP-1 ADCP assay using CH505TF gp140 antigen-conjugated beads as target. Error bars indicate SD of duplicates. Dotted horizontal lines represent phagocytosis values observed in the absence of Ab. **B-E.** Antigen (BaL gp120) **(B)**, FcɣRIIA **(C)**, and FcɣRIIIA **(D)**, FcɣRIIB **(E)**, binding affinity (left, from duplicate assessments of printed antibody spots, bar indicates average/median) and SPR binding profiles (right, raw data in black, kinetic curve fits in red for the ligand concentration series from a representative antibody spot) for VRC01 in IgG1 and IgG3 forms. AU: arbitrary units.

IgG3 variants induced greater ADCP activity than their IgG1 counterparts over a range of effector to target (E:T) ratios and antibody concentrations for both Fab specificities (**Fig 1A**) as defined by a phagocytosis score–the mathematical product of the percentage of phagocytically-active cells and the number of particles phagocytosed per cell [60]. IgG3 exhibited superior phagocytic activity under all conditions tested with the exception of the lowest E:T ratio at the highest 447-52D concentrations, at which both subclasses exhibited equivalent activity. Interestingly, for VRC01, the peak magnitude of phagocytosis was consistently higher for IgG3 as compared to IgG1, but the concentration of antibody at which half-maximal activity was observed appeared fairly consistent. In contrast, peak activity was increased and the concentration of antibody required for half-maximal activity was decreased, by up to an order of magnitude for 447-52D.

We next investigated whether differences in the affinity of IgG1 and IgG3 antibodies for their antigen or FcɣR could account for their differing phagocytic activities. Several studies have evaluated the affinity of human IgG1 and IgG3 for various FcɣR [66–68], and have generally reported either similar binding affinities or slightly higher affinity for IgG3. However, because these interactions can be impacted by post-translational glycosylation of the Fc, affinities to activating FcɣRIIa and FcɣRIIIa and inhibitory FcɣRIIb receptors expressed by THP-1 cells were evaluated to test whether differences in binding affinity could explain the difference in potency. However, in a multiplexed surface plasmon resonance (SPR) assay to define binding affinities between antibodies and antigen (**Fig 1B**) and antibodies against FcɣRs (**Fig 1C and 1D**), only minor differences were observed, suggesting that differences in affinity for either antigen or FcɣR were not likely responsible for the striking differences in phagocytic potency. To further evaluate the possibility that despite consistent intrinsic affinity for antibody, subclass switching could drive differences in antigen recognition capacity in the context of an antigen-bearing particle, antigen beads were incubated with subclass switched antibodies to define the levels of bound antibody. No differences in binding efficiency were observed in these tests (**Figure A in S1 Text**), suggesting that a difference in opsonization capacity was likewise not responsible for the observed activity differences.

### IgG1 and IgG3 hinge regions rather than CH2 and CH3 domains affect phagocytosis

**Fig 2:**
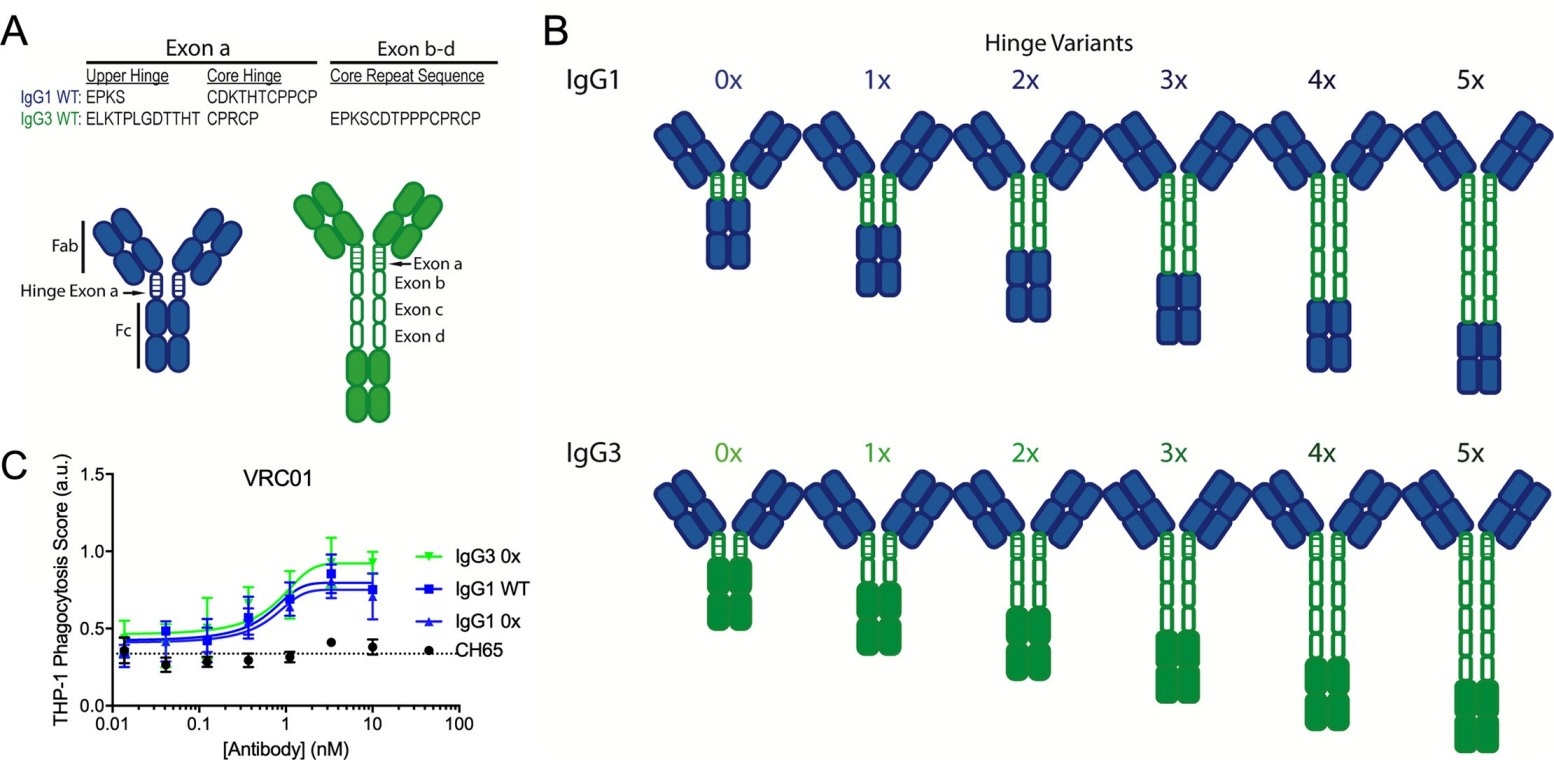
IgG1 and IgG3 hinge variant panel. **A.** Sequences of natural IgG1 and IgG3 hinge exons, noting the upper and core hinge regions encoded by exon a and the core hinge repeat sequences encoded by exons b-d in IgG3. **B.** Schematic of the panel of hinge swapped and extended IgG1 and IgG3 variants. Domains derived from IgG1 and IgG3 are indicated in blue and green respectively. **C.** Phagocytic activity of VRC01 WT IgG1 and 0x forms of IgG1 and IgG3 in the THP-1 ADCP assay against CH505TF gp140 antigen-conjugated beads. Error bars indicate mean and SD of duplicates. Dotted horizontal line represent phagocytosis value observed in the absence of Ab. Phagocytic scores for CH65, an IgG1 Ab specific for hemagglutinin are shown as an additional negative control. Connecting lines indicate curve fit models. AU: arbitrary units.

The striking potentiation in phagocytic activity but lack of differences in antibody binding to either FcɣR or antigen suggested that other factors, such as differences in antibody flexibility and spatial conformations or constraints in the context of ternary interactions with a target particle and effector cell, could explain the observed differences between IgG1 and IgG3. Because the hinge region is a major structurally differentiating factor between the two subclasses, we hypothesized that the extended hinge structure and sequence might account for the enhanced phagocytic activity of IgG3. To evaluate this possibility, a series of VRC01 and 447-52D antibody hinge variants were generated in which the native upper and core hinge sequences (**Fig 2A**) were deleted, swapped, and extended, while flanking CH domains were not modified (**Fig 2B**). First, to define the impact of the IgG3 core hinge repeats, these regions were deleted, resulting in a variant (IgG3 0x) that demonstrated phagocytic activity comparable to IgG1 (**Fig 2C**). Second, to define the impact of the upper hinge, which comprises the linkage between the disulfide-bonded hinge core and the CH1 domain and is somewhat longer in most IgG3 allotypes as compared to IgG1, this region was swapped into the VRC01 IgG1 backbone (IgG1 0x). Interestingly, no difference in phagocytic activity was observed between this variant and unmodified IgG1 (**Fig 2C**), suggesting that the marginally increased length of the IgG3 upper hinge, which in principle could result in different conformational flexibility and reach, was not responsible for the enhanced phagocytic activity of IgG3. Collectively, these results suggested that the IgG3 core hinge region, which for most IgG3 allotypic variants is encoded by three repeated exons, was responsible for enhancing ADCP activity.

Collectively, despite the length and potential disulfide bond differences between the IgG1 and IgG3 exon A, almost no difference in phagocytic activity was observed between IgG1 WT, IgG1 0x, and IgG3 0x. Thus, rather than being associated with the IgG3 CH2 and CH3 domains, the enhanced phagocytic activity of IgG3 may instead be associated with its elongated hinge. Further, the reduced activity that resulted from removing exons B-D raised the possibility that hinge length may be directly associated with increased phagocytosis.

### IgG3 ADCP activity is dependent on hinge length

To more directly test for a relationship between hinge length and phagocytic function, a series of VRC01 and 447-52D IgG3 variants in which the core hinge repeats were deleted or extended was generated, resulting in a panel of antibodies with stepwise variation in hinge length from zero to five core hinge repeats (**Fig 2B**). Structurally, the hinge increases the distance between the Fab and Fc regions and possibly also affects conformational flexibility between these domains. Human IgG3 allotypes exist with one (1x) and two (2x), but mostly commonly three (3x, or wildtype (WT)) core repeats [69, 70]; similarly elongated hinges have not been reported among IgG subclass types in commonly used model organisms. Two extended hinge lengths with four (4x) and five (5x) repeats were also generated in order to test whether extending the hinge was a viable means to further increase phagocytic function.

**Fig 3:**
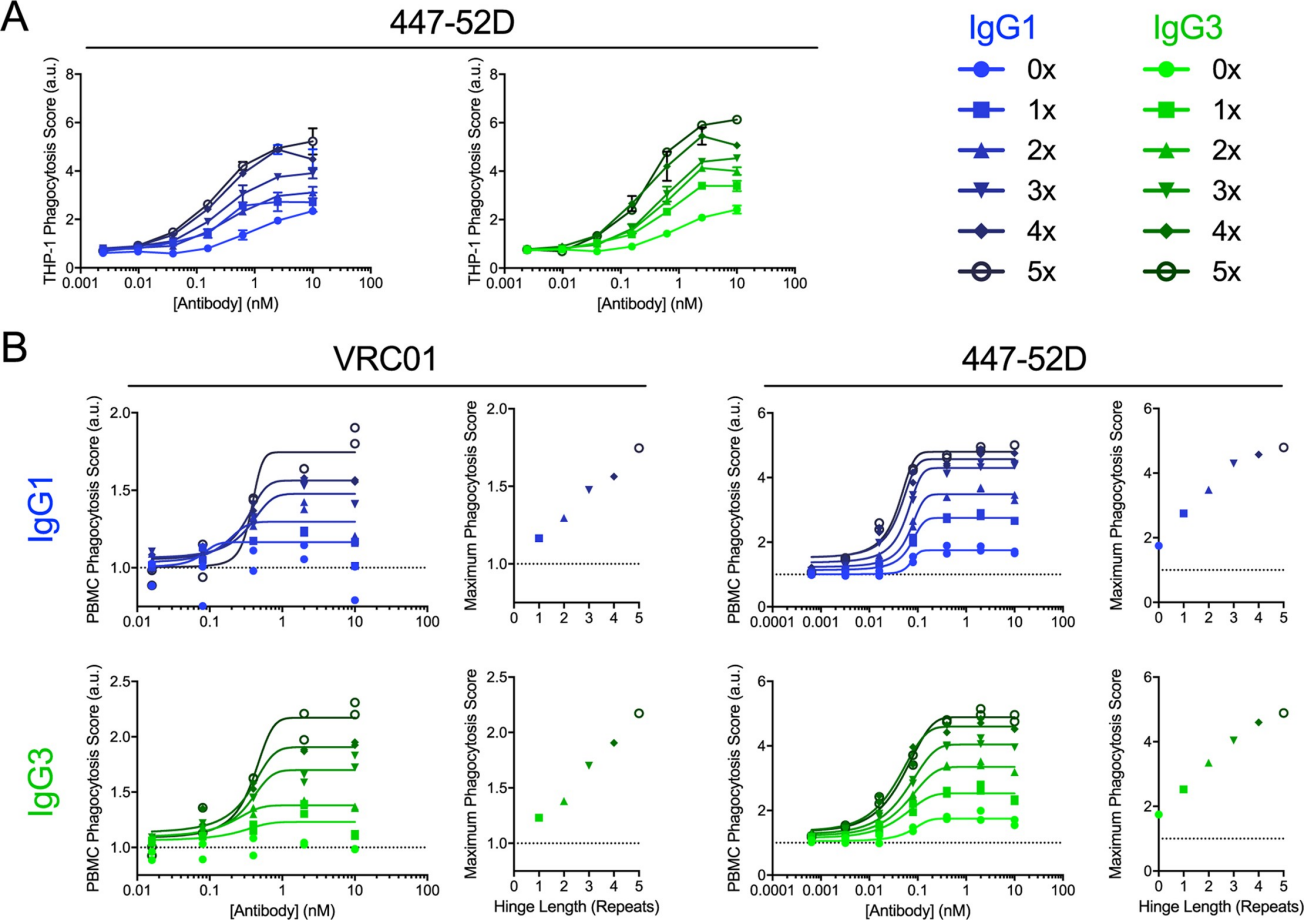
Increasing hinge length increases phagocytic activity. **A.** Phagocytic activity of IgG1 (left) and IgG3 (right) forms of 447–52 in the THP-1 ADCP assay against CH505TF gp140 antigen-conjugated beads. Error bars indicate mean and SD of duplicates. **B.** Phagocytic activity of VRC01 (left) and 447-52D (right) in IgG1 (top) and IgG3 (bottom) in the PBMC ADCP assay against CH505TF gp140 antigen-conjugated beads. Connecting lines indicate curve fit models. Inset scatterplots depict the relationship between hinge length and peak phagocytic score observed from curve fits. Data was collected in duplicate and normalized to a no-Ab control within each plate. Dotted horizontal lines represent phagocytosis values observed in the absence of Ab. AU: arbitrary units.

IgG3 hinge variants generated for 447-52D and VRC01 were assayed using THP-1 as effectors and antigen beads as targets at an E:T ratio of 1:5. A general trend was observed in which phagocytic activity increased with increasing hinge length (**Fig 3A, Figure B in S1 Text**). Consistent with other studies that have modified hinge composition [71, 72], the antibodies tested tolerated hinge engineering. Naturally occurring allotypes (1x, 2x, and 3x) also demonstrated different *in vitro* activity from each other, suggesting the merits of defining whether these genetic differences manifest as a biologically significant difference *in vivo*.

Primary human PMBCs, which may better represent the responses of phagocytes *in vivo*, were used to assay ADCP activity on the panel of hinge variants (**Fig 3B**). PBMCs were gated using FSC/SSC to include monocytes but exclude lymphocytes [73] (**Figure C in S1 Text**). For both Fab specificities tested, a direct correlation between IgG3 hinge length and peak phagocytic activity was observed. Again, as controls for potential impacts on Fab and Fc binding due to hinge modifications, binding affinities for antigen and FcɣR were tested, with similar affinities observed for antigen and FcɣR across subclass specificity-matched hinge variants (**Figure D in S1 Text**). Despite improvement in the ADCP activity of both antibodies, VRC01 remained the weaker inducer at a given hinge length, suggesting that subclass and hinge length alone are not sufficient to predict phagocytic activity.

### The IgG3 hinge can be used to enhance IgG1 effector function

Given the modular nature of the hinge and the prevalence of the IgG1 subclass among therapeutic antibodies, we next evaluated whether the swapping of the IgG3 hinge could be used to improve the phagocytic activity of the IgG1 molecule. An IgG1 backbone hinge variant panel was generated to determine whether a hinge length to activity relationship was apparent in the context of a different Fc backbone. For both VRC01 and 447-52D, increasing hinge length resulted in increased phagocytic activity, and a direct correlation between peak phagocytic score and hinge length was observed (**Fig 3**). Again, affinities were measured to check whether subclass switching altered binding, but no correlations between hinge length and affinity for receptor or antigen were observed (**Figure D in S1 Text**). Notably, 447-52D, which has enhanced ADCP activity relative to VRC01, appeared to benefit less from the fourth and fifth repeats. This observation may imply that a physical limit, perhaps to the number of particles that can be easily endocytosed, is being approached.

### Impact of hinge composition on other antibody functions

**Fig 4:**
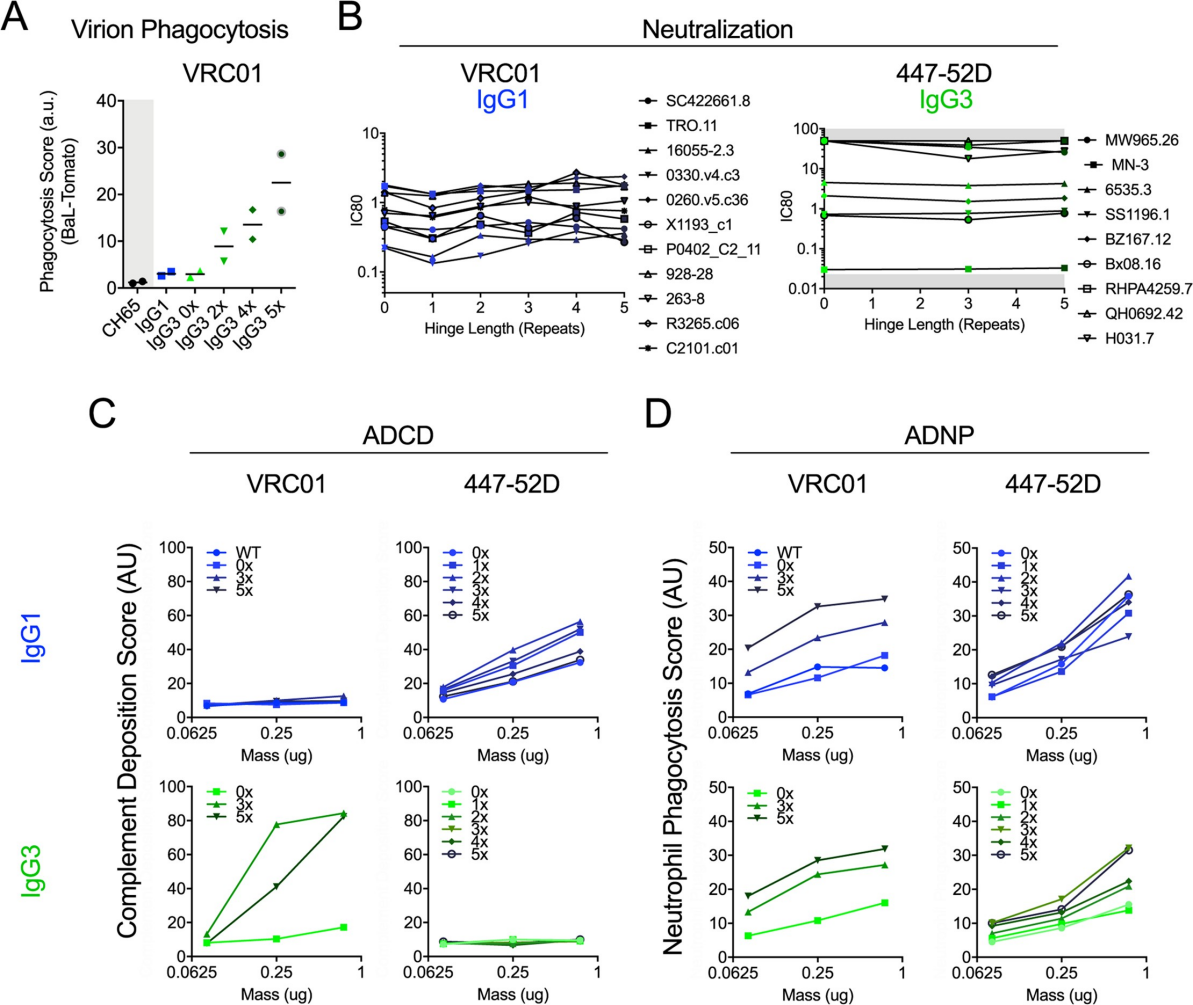
Impact of hinge modification on additional antibody functions. **A.** Uptake of fluorescent virions for IgG1 and IgG3 hinge variant forms of VRC01 and the CH65 control antibody (assessed in duplicate, bar indicates mean). **B.** Neutralization profiles of IgG1 hinge variants of VRC01 (left) and IgG3 hinge variants of 447-52D (right) on selected sensitive viruses. Titrations were performed in duplicate. Gray region indicates the limit of detection. **C-D.** Antibody dependent complement deposition (ADCD) **(C)** and antibody dependent neutrophil phagocytosis (ADNP) **(D)** activity of VRC01 (left) and 447-52D (right) hinge variants in IgG1 (top) and IgG3 (bottom) forms of gp120 MN antigen-coated beads. Data represent means of duplicates. AU: arbitrary units.

Given the striking length-dependent improvement in phagocytosis of the beads used as targets in the ADCP assay, we next investigated the impact of hinge modification on virion phagocytosis. HIV particles are much smaller than the one micron fluorescent beads commonly employed by the ADCP assay; they are expected to present antigenic epitopes in biologically-relevant conformations, orientations, and densities, and may be targets of phagocytes *in vivo*. Select VRC01 variants were used in an assay of HIV virion phagocytosis [13], in which the uptake of fluorescently-labeled virions by THP-1 cells was evaluated. Again, increasing hinge length was associated with increased virion phagocytosis activity (**Fig 4A**), generalizing the observation of hinge length-dependent potentiation of phagocytosis to a particle of a different size and composition.

While monocyte-mediated ADCP of antigen beads was of particular interest based on its association with protection in a number of non-human primate (NHP) vaccine studies [62–64] and in HVTN505 [65], and was therefore of particular interest, we also sought to evaluate the effect of hinge modification on other antibody functions; enhancing ADCP activity via hinge extension may not be desirable if doing so ablates or reduces other beneficial activities. To further define the broader consequences of hinge manipulation, the neutralization, neutrophil phagocytosis, complement deposition, and ADCC capacities of select sets of hinge variant antibodies were determined.

First, we assessed whether neutralization capacity was affected by hinge length variation. The upper hinge of IgG3 is several amino acids extended relative to IgG1, and previous studies have shown that IgG3 exhibits a greater range of accessible Fab:Fab arm angles [33], suggesting potential differences in the ability to bind a target particle bivalently between subclasses. Studies of subclass-switched HIV-specific monoclonal antibodies have generally shown no effect or only slightly enhanced neutralization potency for IgG3 forms, even when hinge disulfide bonds are “unzipped” by virtue of elimination of the cysteine residues proximal to the Fab [52]. However, bispecific antibodies and diFabs with variable length linkers have demonstrated that the length between Fab units can dramatically impact neutralization potency [52, 74]. Studies of polyclonal pools of monovalent and bivalent IgG1 and IgG3 Fabs have likewise suggested that differences in HIV neutralization potency associated with hinge composition can occur [75]. To address this possibility experimentally, a set of sensitive pseudoviruses were selected and concentrations of antibody capable of inhibiting 80% of virus infectivity (neutralization IC80 values) determined across a panel of VRC01 hinge length variant IgG1 antibodies (**Fig 4B**). Hinge length had little to no effect on neutralization potency; most differences observed fell within the range associated with assay variability. Similarly, across a range of variably sensitive viruses, 447-52D in an IgG3 backbone showed no length-dependent change in neutralization potency across different hinge lengths (**Fig 4B**).

Next, the complement deposition properties of hinge variants of IgG1 and IgG3 forms of VRC01 were assessed. The complement system is a highly conserved innate immune pathway. When initiated by antibodies or other factors, the complement cascade can result in covalent modification of target particles with components that are recognized by membrane-expressed complement receptors, and formation of the membrane attack complex and membrane poration. In recent years, the role of Fc-Fc interactions capable of generating antibody hexamers that can efficiently interact with the six-head domains of the complement cascade initiating protein C1q, has become appreciated [7, 76, 77]. Antibody-dependent complement deposition (ADCD) was assessed by measurement of C3d fragment levels on antigen-coated beads following incubation with antibody [17]. Consistent with a prior study that showed strong correlations between IgG3 levels and ADCD activity of polyclonal pools [78], we observed significantly higher activity of IgG3 as compared to IgG1 for VRC01, but the opposite impact of subclass switching on 447-52D (**Fig 4C**). In the context of an IgG1 backbone, hinge modification had a limited impact and inconsistent association with length. In contrast, in the context of the IgG3 backbone, VRC01 with 3x and 5x hinge compositions led to dramatically increased ADCD activity compared to the 0x variant. For antibodies able to drive complement deposition, we observed that for both IgG1 and IgG3 backbones the 2x and/or 3x hinge exhibited highest activity. Though the extent varied, both shorter and longer hinges showed compromised activity. Given the influence of Fc-Fc interactions on ADCD, it is tempting to speculate that while some increase in conformational flexibility and reach is beneficial given the advantage that 3x has relative to 0x, length increases that are too great reduce the likelihood of efficient Fc multimerization as the distance between Fc domains and entropic costs increase due to the greater range of accessible conformations spatially. Further, unlike other activities tested, whether IgG1 or IgG3 CH2 and CH3 domains were present did have a clear impact on function. We observed potentiated ADCD activity for IgG3 relative to IgG1, which is consistent with prior reports [43, 79, 80], though the literature is somewhat conflicting on this point [36, 39, 81]. Therefore, consistent with IgG hexamerization studies, our data support the claim that ADCD activity may be considerably more sensitive than other effector functions to context-specific spatial and conformational factors.

While phagocytes often express a spectrum of FcɣR (FcɣRI, FcɣRIIa, FcɣRIIb, and FcɣRIIIa), including high and low affinity, activating and inhibitory receptors, other phagocytic cells express a more restricted profile of innate immune antibody receptors. Neutrophils, which typically exclusively express FcɣRIIa and FcɣRIIIb, a GPI-linked Fc receptor, are also capable of antibody-dependent phagocytosis, and this activity has also correlated with protection from infection in NHP [62]. Therefore, a neutrophil phagocytosis assay was conducted to determine whether the ADCP activity increase associated with hinge length was generalizable to this effector population. In an adaptation of the ADCP assay that used primary neutrophils as effectors [78], a similar direct relationship between hinge length and phagocytosis was observed in the context of both the IgG1 and IgG3 backbones, for both VRC01 and 447-52D. This observation suggests that phagocytosis by neutrophils, like monocytes, is better mediated when the Fab is more distal to the Fc (**Fig 4D**). Consistent with observations for monocyte ADCP, the IgG3 CH2 and CH3 domains did not appear to impart significant enhancement of neutrophil phagocytosis, as the IgG3 0x variant displayed similar activity as IgG1 WT and IgG1 0x.

Antibody-dependent cellular cytotoxicity (ADCC) mediated by NK cells has been of considerable interest in both infectious disease and oncology. However, prior subclass switching work does not support a strong role for hinge modification in ADCC, as similar activity of IgG3 relative to IgG1 is typically observed [56, 82]. We have previously observed similar induction of NK-degranulation by VRC01 IgG1 and IgG3 [14]. In contrast, a recent study found that hinge extension improved ADCC for one monoclonal antibody, but that this effect was not generalizable across specificities that recognized different epitopes on the same antigen [83]. Recognizing that there are numerous means to assess ADCC, we tested VRC01 hinge variant activity using HIV-1 Infectious Molecular Clone-infected target cells and assessing the reduction in luciferase expression as readout for cellular cytotoxicity [84]. No trend was observed across hinge lengths among the three strains evaluated (**Figure E in S1 Text**). While other factors such as antigen affinity and antigen density have also been associated with modulating ADCC activation [85], the most widely described means to enhanced ADCC activity is via variable fucosylation of the Fc domain. This effect is apparent in the potentiated ability of afucosylated VRC01 IgG1 to induce NK cell degranulation relative to fucosylated IgG1 or IgG3 types, whereas no activity was observed for aglycosylated IgG1 [14]. Antibody glycosylation status was not determined nor controlled for the majority of antibody variants evaluated in this study. Despite having directly assessed FcɣR affinity, differences in glycosylation status still confound our assessment of the impact of subclass and hinge length on ADCC activity in principal.

### Impact of hinge composition on antibody stability

**Fig 5:**
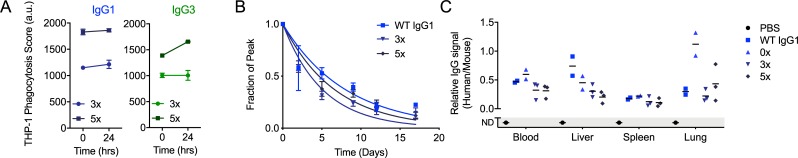
*In vitro* and *in vivo* stability tests. **A.** Serum stability of 447-52D IgG1 and IgG3 in 3x and 5x forms (n = 2, mean and SD plotted) as defined by phagocytic activity of THP-1 cells against JRFL SOSIP antigen-conjugated beads. AU: arbitrary units. **B.** Pharmacokinetic assessment of infused WT VRC01 IgG1, 3x, and 5x variants (n = 2–3, mean and SD are plotted along with 1 phase decay curve fit). **C.** Biodistribution of WT IgG1, 0x, 3x, 5x hinge variants as defined by relative levels of human IgG as compared to mouse IgG detected in each tissue. No human IgG could be detected (ND) in the PBS-infused mouse. Bars indicate means.

Overall, given evidence that IgG hinge modification can dramatically improve phagocytic activity in cell-based assays, we next sought to determine whether hinge elongation might be a promising means to modify antibody activity *in vivo*. To address potential concerns about instability, serum- and thermo-stability testing was performed on select variants. Because circulating serum enzymes could possibly cleave antibodies at the hinge region, other serum factors could interact with and reduce the benefits of the longer hinge, or the molecule might simply have compromised thermal stability, we exposed select hinge length variants (3x and 5x forms of 447-52D) to 10% human serum over 24 hours at 37°C. The phagocytic activities of untreated and serum-exposed antibody toward antigen-conjugated beads were indistinguishable; neither 3x nor 5x variants exhibited a loss of activity under these conditions (**Fig 5A**).

While the previous result was encouraging, there are a multitude of modes of protein clearance possible *in vivo*. The pharmacokinetic (PK) profiles of VRC01 WT IgG1 and 3x and 5x hinge variants in the IgG1 backbone were evaluated in mice to allow comparison of clearance *in vivo*. Serum antibody levels were measured longitudinally starting at one day post intravenous injection to determine the impact of hinge modification on antibody PK. Though group sizes were small, similar plasma decay profiles were observed across the set of variants tested, suggesting that the hinge is not substantially involved in antibody recycling or clearance *in vivo* (**Fig 5B**).

Differences in biodistribution could also result from hinge modification, as antibodies passively diffuse and are actively transported to peripheral tissue. To address the possibility that hinge length may impact the ability of antibodies to localize to diverse tissues, select organs were harvested and antibody levels measured. While these experiments were not powered to resolve fine differences among variants, relative to WT, elevated levels of the 0x hinge variant were observed in the lung. In terms of trends across hinge length, antibodies with decreased hinge length showed greater accumulation in liver, and potentially in blood. In other tissues, strict correlations with hinge length were not found, suggesting that hinge length modification did not predictably result in highly divergent biodistribution profiles (**Fig 5C**). Further evaluation, however, is necessary to define whether subtle distinctions may exist between variants. In sum, *in vitro* and *in vivo* stability and localization data support further consideration of hinge modified antibodies.

## Discussion

Antibody function is defined by the combination of antigen recognition via the Fab region, receptor engagement via the Fc region as well as the hinge region, which bridges the Fab and Fc. In the setting of anti-viral antibodies, subclass switching and hinge engineering experiments evaluating the neutralization potency of HIV-specific antibodies have demonstrated that hinge composition can [52, 86], but does not always [87, 88] impact neutralization capacity. We found that the neutralization activity of VRC01 and 447-52D were not altered by hinge length. However, Richardson et al. find a modest but reproducible effect on the neutralization potency of CAP256 results from hinge modification [89]. Collectively, these data suggest that the impact of hinge composition varies across both different antibodies as well as across different viruses. While many explanations are possible, it is tempting to speculate that differences in the ability to interact avidly with virions form part of the basis for these observations. Consistent with this possibility, others have observed strain and antibody dependent differences in the neutralization potency of monovalent Fab versus bivalent IgG [74, 75]. Careful experiments with homologous and heterologous Fab linked by DNA have defined the importance of distance between antigen binding sites and ability to bind bivalently to neutralization potency [74]. Experiments evaluating the neutralization potency of bispecific antibodies, including those with extended and “unzipped” hinges provide further support for this hypothesis [90]. Non-native molecules such as these have shown orders of magnitude improvements in potency and breadth may result from avid binding, but also suggest that this landscape is rather rugged. Perhaps then, it is no surprise that modest and sporadic changes to neutralization activity have been observed among natural antibody types.

In contrast, as IgG2 and IgG4 antibodies have compromised effector function compared to IgG1 and IgG3, subclass switching has dramatic and generally predictable effects on the effector function of antiviral antibodies, as would be expected from their differing affinities for FcɣR [67]. Between IgG1 and IgG3, however, it has now been observed that the IgG3 subclass is associated with considerably enhanced phagocytic activity across multiple studies for multiple antibodies, including VRC01, 447-52D, CAP256, CH31, CH27, CH28, HG107, 7B2, 2158, and 1361 [12–14, 89]. Here, these observations are extended to include two antibodies that recognize distinct viral epitopes and have differing phagocytic activities in their IgG1 forms. For these antibodies, improved phagocytic activity was not associated with altered monovalent or avid antigen recognition, or with potentiated FcɣR binding. Instead, hinge swapping and engineering experiments showed that improved phagocytic potency could be attributed to the composition of the IgG3 hinge region. Transplantation of the IgG3 hinge into an IgG1 backbone transferred the phagocytosis enhancement phenotype. Further, stepwise deletion and extension of hinge repeats reduced and improved phagocytic activity, respectively. These phenotypes were consistent at varying ratios of effector cells and target particles, whether cell lines or primary phagocytes were used as effectors, and whether fluorescent antigen beads or viral particles were used as targets. The direct relationship between hinge length and phagocytosis may also hold for other specificities, as Richardson et al. now also demonstrate for CAP256 [89]. Collectively, this data points toward hinge modification as an additional tool for antibody engineering, and provides a new rationale to investigate IgG3 responses and allotypic variation in hinge length in the context of cohort studies.

While somewhat less studied than NK cell-mediated effector functions, antibody-dependent phagocytosis can be a predominant mode of action for anti-cancer antibodies [91], in various infectious diseases [92–95], and, relevant to the HIV-specific antibodies evaluated here and in Richardson et al., has been associated with resistance to HIV infection in the nonhuman primate challenge model [62–64] as well as in HVTN505 [65]. To this end, associations between IgG3 responses and improved infectious disease outcomes are numerous and varied [15, 18, 19, 65, 96–98]. Our observations suggest that the presence of long-hinged IgG3 antibodies, as commonly observed early in an immune response or vaccination series [99, 100] can drive a strong phagocytic response, which may be beneficial both for sequestering pathogens as well as further stimulating immunity through enhancing antigen presentation. In conclusion, this work defines differences in antibody effector function profiles conferred by the hinge region, and finds that hinge length correlates with the stimulation of phagocytes. The functional distinctions observed for IgG3 associated with its unique hinge composition have potential implications for both clinical interventions via vaccination and monoclonal therapies.

## Materials and methods

### Antibody cloning, expression, and purification

The antibodies used for this work were generated recombinantly. Separate pCMV vectors encoding the VRC01 IgG1 heavy and light chain sequences [57] were used as a templates for the variable regions (NIH AIDS Reagent Resource Program). The 447-52D heavy and light chain variable sequences were derived from published amino acid sequences and synthesized as IDT gBlock for cloning onto hinge length backbones. The InvivoGen pFUSE-hIgG3-Fc served as a template for the IgG3 hinge as well as the Fc sequence for IgG3. An IgG3 hinge variant was first synthesized by cloning the VRC01 IgG1 Fab region onto the hinge and Fc of the pFuse vector. The various hinge lengths used were synthesized as codon optimized IDT gBlocks and swapped into the VRC01 IgG3 variant; sequence homology designed in the gBlocks in the CH1 and lower hinge regions allowed for easy insertion of the hinges. The same homology was used to transfer the hinge length sequences from the IgG3 vectors into the original VRC01 IgG1 vector to generate the IgG1 family of hinge variants. More specifically, a BstEII site located in the CH1 domain and an AhdI site located in the lower hinge exists within all constructs used (**Table A in S1 Text**). The 447-52D [59] IgG1 and IgG3 hinge families was cloned using a similar strategy which began by swapping the variable region out of the VRC01 IgG3 before modifications to the hinge regions. Successful cloning was verified using Sanger sequencing (Genewiz) to check for correct sequence.

Antibodies were expressed by co-transfection of heavy and light chain plasmids in Expi293 HEK cells (Thermo Fisher) according to the manufacturer’s instructions. Seven days after transfection, cultures were spun at 3000 x g for 30 minutes to pellet the cells, and supernatants were filtered (0.22 μm). IgG was affinity purified using a custom packed 5 mL protein G column with a retention time of 1 minute (ie. 5 mL/min) and eluted with 100 mM glycine pH 3, which was immediately neutralized with 1 M Tris buffer pH 8. Eluate was then concentrated to 2.5 mL for size exclusion chromatography on a HiPrep Sephacryl S-200 HR column using an AktaPure FPLC at a flow rate of 1 mL/min of sterile PBS. Fractions containing monomeric IgG were pooled and concentrated using spin columns (Amicon UFC903024) to approximately 1 mg/mL of protein and either used within a week or aliquoted and frozen at -80°C for later use.

### Phagocytosis assays

A modified ADCP assay [60, 101] was conducted using the THP1 (ATCC TIB-202) cell line. Briefly, fluorescent antigen-coupled beads were generated using Spherotech SPHERO Carboxyl Fluorescent Particles (CFP-0852-2) 0.85 μm medium intensity beads. To start, 200 μL of beads (approximately 3x10^8^ beads) were washed twice with 500 μL coupling buffer (50 mM MES pH 6.0). Wash steps consisted of room temperature (RT) centrifugation for 10 minutes at 5000 x g, aspiration, and resuspension by pipetting and vortexing. Beads were activated for 20 minutes at RT with end-over-end (EOE) rotation in 500 μL of coupling buffer with 5 mg/ mL EDC (1-ethyl-3-(3-dimethylamino) propyl carbodiimide, hydrochloride) and 5 mg/mL Sulfo-NHS (N-Hydroxysulfosuccinimide). Following two washes with coupling buffer, 10–100 μg of antigen at 1 mg/mL was prepared and added to the beads and volume was brought up to 1 mL total with coupling buffer. Antigen was coupled for 1 hour at RT with EOE mixing. After antigen coupling, beads were pelleted and washed twice in 500 μL PBS + 1% FBS. After washing, beads were resuspended in 1 mL of PBS + 1% FBS and sonicated in a VWR Ultrasonic Cleaner (Cat# 97043–960) for 1 minute before blocking was allowed to occur for 2 hours at RT or overnight at 4°C with EOE mixing. Blocked beads were washed twice with PBSF (PBS + 0.1% BSA), and resuspended to a final volume of 1 mL for storage at 4°C. Beads were mixed by vortexing and sonication before use in phagocytosis assays. Phagocytosis assay results are generally reported for CH505TF gp140-conjugated beads. Independent of the capture antigen, consistent activity patterns have been noted across hinge and subclass variants. For example, activity of VRC01 in IgG3 form has been reported to be similarly enhanced relative to IgG1 in the THP-1 phagocytosis assay when JRFL SOSIP target beads are used [14]. Further, the activity of myeloma-derived IgG3 relative to myeloma IgG1 shows the same enhancement in the THP-1 assay when an anti-IgG target bead is used [56].

THP-1 monocyte cells were cultured in growth media (RPMI 1640, L-glutamine, 10% FBS, non-essential amino acids, and sodium pyruvate) to greater than 500,000 cells/mL then diluted to 100,000 cells/ml. Fluorescent antigen beads were spiked into the cell dilution at the appropriate E:T ratio) and 200 μL of that mixture was transferred to a 96-well tissue culture plate (Corning CLS3595). A volume of 50 μL of antibody diluted in growth media was added to each well for a final volume of 250 μL and plates were transferred to a 37°C, 5% CO_2_ incubator for four hours of incubation to allow for phagocytosis. At the end of the incubation, the assay plate was centrifuged for 5 minutes at 500 x g and washed with cold PBS, which was repeated a second time to remove residual serum which could interfere with fixation. A 4% paraformaldehyde in PBS solution was made fresh and 100 μL of fixative solution was added to the wells and allowed to occur for 20 minutes in at the dark at RT. The fixation reaction was then quenched with an equal volume of 5% FBS in PBS solution and cells were centrifuged at 500 x g for 5 minutes. The pelleted cells were resuspended with 100 μL of PBSF (PBS, 0.1% BSA) and immediately read on a MACSQUANT (Miltenyi) flow cytometer (MACSQuantify 21 software) or stored at 4°C and protected from light for analysis up to 24 hours afterwards.

Data analysis was conducted using Flowjo VX software. Phagocytosis scores were calculated by multiplying the percentage of fluorescent (bead positive) cells by the mean fluorescent intensity (MFI) of the positive cells, often referred to as an integrated mean fluorescent intensity (iMFI). These parameters are illustrated independently in **Figure F in S1 Text**. Cells were identified via gating on FSC/SSC, and gating for bead positive cells was defined based on negative controls. In some cases, normalized phagocytic scores (iMFI_condition of interest_/iMFI_no antibody control_) are reported.

PMBC phagocytosis was conducted similarly to THP1 phagocytosis. Frozen aliquots were obtained and thawed as previously described [102]. Cells were removed from liquid nitrogen storage and immediately thawed using a 37°C bead bath. Cells were washed with 14 mL pre-warmed (37°C) RPMI 1640 + L-glutamine (Thermo Fisher 11875093) supplemented with 10% FBS and 100 u/ml Benzonase (EMD 70664–3). Cells were centrifuged at 400 x g for 10 minutes, after which supernatant was removed, and cells were resuspended in 10 mL of RPMI 1640 + 10% FBS for counting. Total cells were counted using a flow cytometer and used at a ratio of 1:1 total cell to bead ratio for phagocytosis. The phagocytosis assay was then performed as described above. Flow analysis of the PMBCs was conducted based on FSC and SSC gating to identify monocytes (**Figure C in S1 Text**).

Virion phagocytosis was conducted as previously described [13, 103, 104]. Briefly, fluorescently labelled virions [105, 106] were incubated with 25 μg/ml antibody for 2 hours at 37°C. Subsequently, primary monocytes were added to the immune complexes, spinoculated together for 1 hour at 4°C, then incubated at 37°C for 1 hour before washing and fixation. 10,000 CD4-blocked primary monocytes, isolated from human PBMCs via elutriation, were used per well. Fluorescent virus uptake was quantified via flow cytometry. Normalized phagocytic scores were reported as described above.

Neutrophil-mediated phagocytosis (ADNP) was performed in an adaptation of the ADCP assay as previously described [62]. Briefly, fluorescent, streptavidin microspheres were coated with chemically biotinylated gp120 YU2 (Immune Technology) for 2 h at 37°C, washed with 0.1% BSA in PBS, and incubated with dilute antibody for 2 h at 37°C. White blood cells (WBCs) were isolated from healthy donor blood anticoagulated with acid citrate dextrose by ACK lysis of red blood cells. 5x10^4^ WBCs were added to washed, antibody-opsonized beads and incubated for 1 h at 37°C prior to staining with anti-CD66b-Pacific Blue (Biolegend). Cells were then fixed with 4% paraformaldehyde solution and analyzed on a flow cytometer. A phagocytic score was derived as an integrated MFI by multiplication of the fraction of neutrophils that phagocytosed one or more opsonized beads by the MFI of this population.

### Other effector functions

Antibody dependent complement deposition was evaluated by detection of C3b on antigen-coated beads [107]. Briefly, fluorescent, streptavidin microspheres were coated with chemically biotinylated gp120 MN (Immune Technology) for 2 h at 37°C, blocked with 5% BSA in PBS, and incubated with diluted antibody for 2 h at 37°C. Guinea pig complement (Cedar Lane) diluted into veronal buffer with 0.1% gelatin (1:60 dilution) was added to washed, antibody-opsonized beads and incubated for 20 min at 37°C. The beads were washed with 15 mM EDTA in phosphate-buffered saline (PBS), and complement deposition detected by flow cytometry by staining with Anti-C3b-FITC (MP Biomedicals).

NK degranulation was assessed by CD107a expression on the NK-92 cells line after incubation with JRFL SOSIP-coated ELISA plates in the presence of antibody, as previously reported [14]. ADCC was assessed utilizing a modified version of previously published luciferase-based assay [108]. Briefly, CEM.NKR_CCR5_ cells [109] were used as targets for ADCC luciferase assays after infection with HIV-1 infectious molecular clones. Cryopreserved PBMC from HIV-seronegative subjects were used as effector cells at an effector-to-target cell ratio of 30:1. Target and effector cells were plated in opaque 96-well half-area plates and co-cultured with serial dilutions of antibodies starting with 50 μg/ml and sequential 1:5 dilutions. Co-cultures were incubated for 6 hours at 37°C in 5% CO_2_. The final readout was the luminescence intensity generated by the presence of residual intact target cells that have not been lysed by the effector population in the presence of ADCC-mediating antibodies. The percentage of killing was calculated using the formula:

$$\%\;specific\;killing=\frac{RLU\;of\;target\;and\;effector\;well–\;RLU\;of\;test\;well}{RLU\;of\;target\;and\;effector\;well}*100,$$

Where RLU is relative light units. In this analysis, the RLU of the target plus effector wells represents spontaneous lysis in the absence of any source of antibody.

Minimally, a no antibody condition was included as a negative control in each run of each functional assay, but in some cases, negative control antibodies, such as the influenza-specific CH65 [110] antibody were also used. “Wildtype” VRC01 and 447-52D IgG1 and IgG3 antibodies, and or polyclonal IgG purified from HIV+ donors (HIVIG) were considered positive controls.

### Binding and affinity to FcɣR and antigen

Affinity data was collected via surface plasmon resonance imaging (SPRi) using a MX96 (IBIS Technologies) and previously published protocols [56, 66]. Briefly, discrete regions on a CMD200M sensorchip (XanTec bioanalytics) were activated with 3 mM sulfo-NHS and 10 mM EDC formulated in 10 mM MES (pH 5.0) applied by continuous flow microspotter (Carterra). Immediately following activation, antibody of interest formulated at 100 or 200 nM in 10 mM sodium acetate (pH 5.0) was flowed over the active regions. The entire sensor surface was then quenched with 1 M ethanolamine. Binding signals across a seven point dilution series of recombinant ligands, sequentially flowed over the chip at up to a 25 μM final concentration for FcɣR, which were produced as described [111], or 1 μM for antigen gp120 BaL (NIH AIDS Reagent Program) were collected. The resulting signals were double referenced in SprintX (IBIS Technologies) by subtracting signal from the linked interspot regions adjacent to the regions of interest and the blank injection immediately preceding the lowest concentration of each receptor. Kinetic curves were fit to the results in Scrubber 2 (BioLogic Software) and manually screened for goodness of fit and sufficient signal. K_D_s were calculated by defining the start and stop of the injections and fitting k_d_s which were then fixed. k_a_ and bulk RI was floated and K_D_s then automatically calculated from those fits, which were manually screened to ensure goodness of fit for quality sensorgrams.

Antibodies were tested for their opsonizing capacity to against HIV antigen. JRFL SOSIP was coupled to Luminex beads as described above. Variants VRC01 IgG1 0x, 3x, and 5x were titrated against those capture beads and goat anti-human IgG Fc, multi-species SP ads-PE (Southern Biotech 2014–09) was used to detect the presence of antibody on the surface.

### Neutralization assay

Antibodies were tested for their neutralization activity using methods previously described [112, 113] Briefly, the amount of antibody needed to result in 80% suppression of the HIV-1 Tat-regulated luciferase signal from TZM-bl cells upon infection with HIV envelope pseudotyped viruses was defined. Viruses tested were selected based on known sensitivity to either VRC01 of 447-52D from historical data.

### Stability tests

*In vitro* stability was tested using a functional readout after incubation of dilute antibody at 37°C with 10% human serum. Whole blood was centrifuged at 500 x g to pellet immune cells. Supernatant was collected and rapidly frozen at -80°C to lyse cells. Monoclonal hinge variants were titrated and diluted in 10% human serum and RPMI 1640 and incubated at 37°C in a C1000 Touch Thermal Cycler for 24 hours. Titrations were used in a THP-1 phagocytosis assay to read out functionality post incubation.

### Ethics statement

Animal experiments were conducted according to protocols approved by the IACUC of The Geisel School of Medicine at Dartmouth under protocol number 00002151(a). All housing and animal care conforms to the “Guide for the Care and Use of Laboratory Animals” and the Public Health Service Policy on Humane Care and Use of Laboratory Animals.

### In vivo infusions

Mice were housed at Dartmouth’s Center for Comparative Medicine and Research. Twelve C57BL/6J mice (The Jackson Laboratory) bred in house, of both sexes at 6–8 weeks of age were used for pharmacokinetic and biodistribution experiments. PBS vehicle control (n = 1), VRC01 IgG1 (n = 2), and VRC01 IgG1 0x, 3x, and 5x (n = 3 each) were administered with at least one male and one female per group. Antibody (10 μg in 100 μL of PBS) was administered via tail vein injection under isofluorene sedation under a heat lamp. Samples (50–100 μL) were collected via cheek bleed every other day beginning at 16 h post injection. Harvested blood was chilled immediately to -80°C to lyse cells. At the conclusion of the final time point, mice were sacrificed per IACUC protocols and cardiac perfusion with PBS was performed to remove serum antibodies from peripheral tissue. Lungs, spleen, and liver were harvested and homegenated with a tissue blender (Omni International) in PBS with protease inhibitor (Roche) and stored at -80°C for further analysis.

### Serum antibody quantitation

A multiplexed bead assay was employed to detect and quantify human, mouse, and gp120-specific antibodies. Fluorescently-coded microspheres were amine-coupled to capture reagents: anti-mouse IgG (Southern Biotech 1010–01), F(ab’)2-goat anti-human IgG Fc (ThermoFisher A24478), or gp120 (CCH5067, JRCSF, or gp120MN; provided by the NIH AIDS Reagent program), as previously described. [114]. Bead-bound antibodies were detected with Goat anti-mouse Ig, Human ads-PE (Southern Biotech 1010–09) Goat Anti-Human IgG Fc, Multi-Species SP ads-PE (Southern Biotech 2014–09), as appropriate; the species-specificity and lack of cross-reactivity of these reagents in this assay was defined previously. Collected whole blood was first centrifuged at 500 x g at RT to pellet cells for 15 minutes, and the supernatant diluted to a non-saturating range in assay buffer (PBS, 0.1% BSA, 0.05% Tween 20) prior to incubation, staining, and detection of signal on a FlexMAP 3D (Luminex) using xPONENT software, as previously described [115].

### Data analysis

Where shown, non-linear regression curve fitting (sigmoidal, 4PL, least squares fit) was conducted for antibody titrations. Lower bound values were set to the values observed for negative controls (beads and cells in the absence of antibody). Upper values, log IC_50_, and Hill slope were calculated using Graphpad Prism (Version 6.07).

Serum levels of passively infused antibodies over time were modeled using a one phase decay fit, with models constrained at peak (measured one day post-infusion) and plateau values of 1 and 0, respectively, using Graphpad Prism (Version 7.0d).

## Supporting information

S1 Text Supplementary figures and tables. (DOCX)
