# Supplementary material for: Hinge length contributes to the phagocytic activity of HIV-specific IgG1 and IgG3 antibodies
Source: PLoS Pathog. 2020 Feb 24;16(2):e1008083. doi: 10.1371/journal.ppat.1008083 (PMC7058349; doi:10.1371/journal.ppat.1008083)
Supplement: S1 Text — (DOCX) [file ppat.1008083.s001.docx]

**S1 Text**

| **Supporting Figures** |  |
| --- | --- |
| S1 Figure A | Opsonization of JRFL SOSIP-conjugated beads by selected hinge variants |
| S1 Figure B | THP-1 phagocytosis data for VRC01 hinge variant panel |
| S1 Figure C | Gating strategy used for the PBMC phagocytosis assay |
| S1 Figure D | Extended receptor and antigen affinity data generated from multiplexed SPR |
| S1 Figure E | ADCC data for select hinge variants of VRC01 |
| S1 Figure F | Individual components of the phagocytosis score plotted separately |
| **Supporting Tables** |  |
| S1 Table A | DNA Sequences |

**S1 Figure A**. Opsonization of JRFL SOSIP-conjugated beads by selected hinge variants.

**S1 Figure B**. Phagocytosis activity of VRC01 IgG3 hinge variants in THP-1 assay against CH505TF gp140 antigen-conjugated beads. AU: arbitrary units.


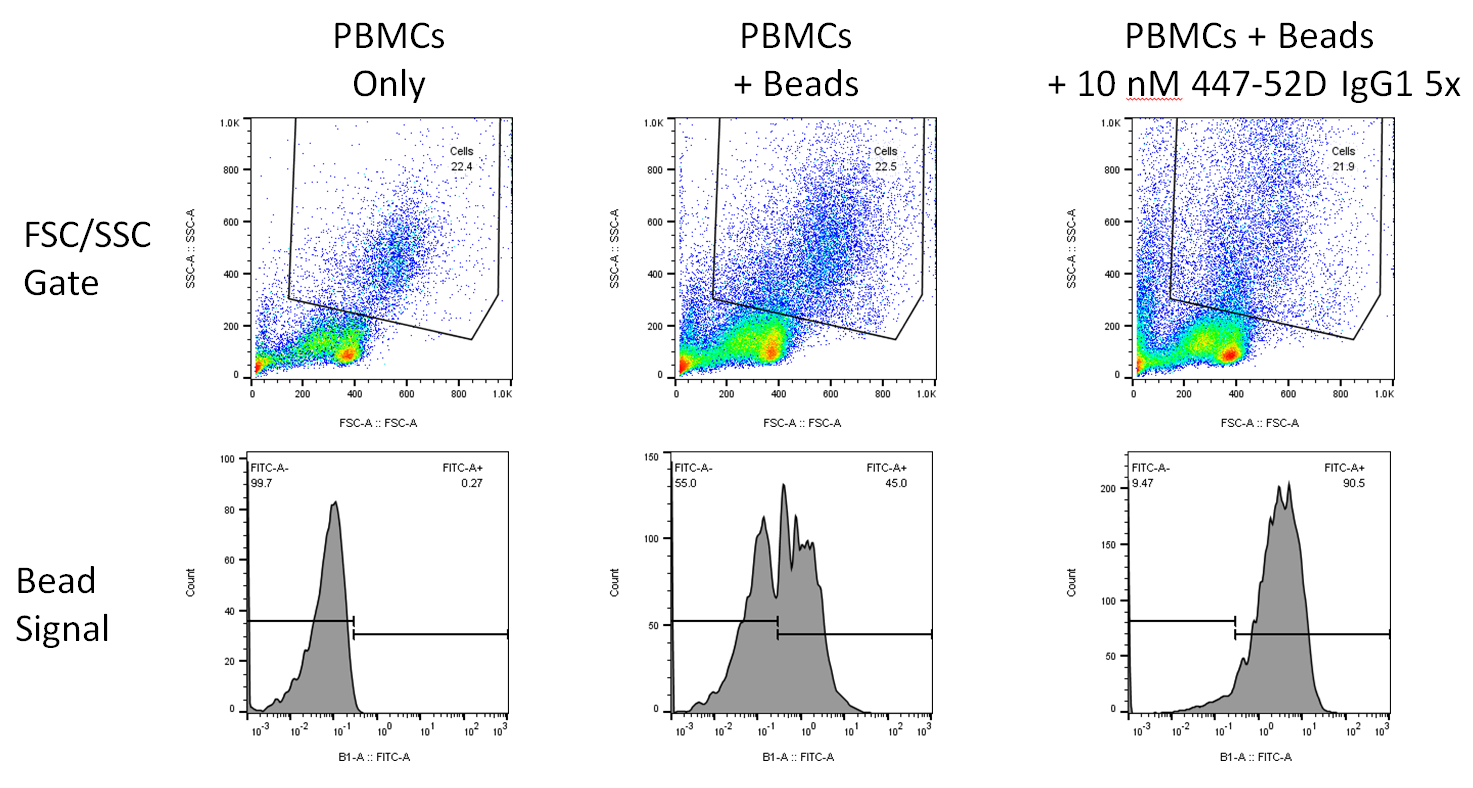


**S1 Figure C**. Gating strategy used for the PBMC phagocytosis assay. The FSC/SSC gate was drawn to exclude non-phagocytosed beads (low FSC, high SSC) as well as lymphocytes (low FSC, low SSC). Along with monocytes, granulocytes were captured by the gating strategy employed, which was also designed to also account for gain in SSC for cells phagocytosing beads. As a negative control, PMBCs alone (left) were used to draw a gate for bead positive cells. As observed for THP-1 cells, PBMC-derived cells naturally phagocytose some CH505TF gp140 antigen-conjugated beads even in the absence of antibody (center) which were used to normalize the amount of antibody-dependent phagocytic activity (right).

**S1 Figure D**. Extended receptor and antigen affinity data generated from multiplexed SPR.

**S1 Figure E**. ADCC activity of select VRC01 hinge variants. Peak ADCC activity mediated by VRC01-hinge variants against HIV-1 Infectious Molecular Clones using CEM.NKR cells at targets and PBMCs from seronegative donor as effectors at an E:T ration of 30:1. Virus strain is indicated in color.

**S1 Figure F**. Individual components of the phagocytosis score plotted separately. Percentage of cells which phagocytose one or more beads (left) and MFI of bead positive cells (right) across a titration of antibody for 447-52D IgG1 hinge variants against CH505TF gp140 antigen-conjugated beads using PBMC as effectors. Connecting lines indicate curve fit models. Dotted horizontal line represents activity observed in the absence of Ab. AU: arbitrary units.

**S1 Table A**. DNA Sequences

| **Hinge** | **Sequence (5’ 🡪 3’)** |
| --- | --- |
| 0x | GAGCTCAAAACCCCGCTTGGGGATACCACTCACACATGCCCCAGATGTCCA |
| 1x | GAACTGAAGACACCCTTGGGCGATACAACTCATACTTGCCCACGATGCCCTGAGCCAAAATCCTGCGACACACCGCCACCCTGCCCCCGCTGTCCA |
| 2x | ACCCCTTTGGGAGACACAACCCATACCTGCCCCCGCTGTCCCGAGCCTAAATCCTGCGACACACCCCCTCCTTGTCCCAGGTGTCCTGAACCCAAAAGCTGCGACACACCTCCTCCATGTCCGCGCTGCCCC |
| 3x | GAATTGAAGACACCTCTCGGCGACACTACTCACACATGCCCAAGATGCCCAGAGCCTAAGTCCTGCGACACCCCTCCTCCCTGTCCTAGATGCCCTGAGCCAAAGTCTTGCGATACGCCTCCACCCTGCCCTCGGTGTCCTGAGCCTAAATCATGCGATACCCCACCACCATGTCCTCGCTGCCCC |
| 4x | GAACTCAAAACACCTCTGGGAGATACTACGCACACTTGCCCCAGGTGTCCAGAACCCAAGTCATGCGACACCCCTCCGCCATGTCCCAGGTGCCCCGAGCCAAAGAGCTGTGATACACCTCCTCCATGTCCCAGGTGTCCAGAGCCCAAGTCTTGTGATACACCGCCCCCTTGCCCACGCTGTCCTGAGCCCAAGTCCTGCGATACACCCCCACCATGTCCGAGGTGCCCT |
| 5x | GAACTTAAAACCCCCTTGGGCGACACGACACATACATGCCCCCGCTGCCCCGAGCCAAAGTCATGTGATACTCCTCCCCCATGCCCGAGGTGTCCAGAACCTAAGTCATGCGACACTCCTCCCCCCTGCCCCAGGTGCCCTGAGCCAAAGTCTTGTGACACACCGCCGCCCTGCCCACGCTGTCCTGAGCCTAAGAGCTGCGATACACCACCTCCCTGTCCTCGATGTCCGGAACCCAAATCTTGTGATACCCCTCCTCCTTGCCCCCGGTGCCCT |
|  |  |
| VRC01 V_h_ | CAGGTGCAGCTGGTGCAGTCTGGAGGTCAGATGAAGAAGCCTGGCGAGTCGATGAGAATTTCTTGTCGGGCTTCTGGATATGAATTTATTGATTGTACGCTAAATTGGATTCGTCTGGCCCCCGGAAAAAGGCCTGAGTGGATGGGATGGCTGAAGCCTCGGGGGGGGGCCGTCAACTACGCACGTCCACTTCAGGGCAGAGTGACCATGACTCGAGACGTTTATTCCGACACAGCCTTTTTGGAGCTGCGCTCGTTGACAGTAGACGACACGGCCGTCTACTTTTGTACTAGGGGAAAAAACTGTGATTACAATTGGGACTTCGAACACTGGGGCCGGGGCACCCCGGTCATCGTCTCA |
| VRC01 V_l_ | GAAATTGTGTTGACACAGTCTCCAGGCACCCTGTCTTTGTCTCCAGGGGAAACAGCCATCATCTCTTGTCGGACCAGTCAGTATGGTTCCTTAGCCTGGTATCAACAGAGGCCCGGCCAGGCCCCCAGGCTCGTCATCTATTCGGGCTCTACTCGGGCCGCTGGCATCCCAGACAGGTTCAGCGGCAGTCGGTGGGGGCCAGACTACAATCTCACCATCAGCAACCTGGAGTCGGGAGATTTTGGTGTTTATTATTGCCAGCAGTATGAATTTTTTGGCCAGGGGACCAAGGTCCAGGTCGACATTAAA |
| 447-52D V_h_ | GAGGTGCAGCTGGTGGAGTCTGGGGGAGGCTTGGTAAAGCCTGGGGGGTCCCTCAGACTCACCTGTGTAGCCTCTGGTTTCACGTTCAGTGATGTCTGGCTGAACTGGGTCCGCCAGGCTCCAGGGAAGGGGCTGGAGTGGGTCGGCCGTATTAAAAGCAGAACTGATGGTGGGACAACAGACTACGCTGCATCCGTGAAAGGCAGATTCACCATCTCAAGAGATGACTCAAAAAACACGCTATATCTGCAAATGAATAGCCTGAAAACCGAGGACACAGCCGTTTATTCCTGCACCACAGATGGTTTTATTATGATTCGGGGAGTCTCCGAGGACTACTACTACTACTACATGGACGTTTGGGGCAAAGGGACCACGGTCACGGTCTCCTCA |
| 447-52D V_l_ | CAGTCTGTGTTGACGCAGCCGCCCTCAGTGTCTGCGGCCCCAGGACAGAAGGTCACCATCTCCTGCTCTGGAAGCAGCTCCAACATTGGGAATAATTATGTATTGTGGTACCAGCAGTTCCCAGGAACAGCCCCCAAACTCCTCATTTATGGCAATAATAAGCGACCCTCAGGGATTCCTGACCGATTCTCTGGCTCCAAGTCTGGCACGTCAGCCACCCTGGGCATCACCGGACTCCAGACTGGGGACGAGGCCGATTATTTCTGCGCAACATGGGATAGCGGCCTGAGTGCTGATTGGGTGTTCGGCGGAGGGACCAAGCTGACCGTCCTAAGT |
| CH1 | TCACCGTCGACCAAGGGCCCATCGGTCTTCCCCCTGGCACCCTCCTCCAAGAGCACCTCTGGGGGCACAGCGGCCCTGGGCTGCCTGGTCAAGGACTACTTCCCCGAACCGGTGACGGTGTCGTGGAACTCAGGCGCCCTGACCAGCGGCGTGCACACCTTCCCGGCTGTCCTACAGTCCTCAGGACTCTACTCCCTCAGCAGCGTGGTGACCGTGCCCTCCAGCAGCTTGGGCACCCAGACCTACATCTGCAACGTGAATCACAAGCCCAGCAACACCAAGGTGGACAAGAAAGTT |
| IgG1 Lower Hinge-CH2-CH3 | GCACCTGAACTCCTGGGGGGACCGTCAGTCTTCCTCTTCCCCCCAAAACCCAAGGACACCCTCATGATCTCCCGGACCCCTGAGGTCACATGCGTGGTGGTGGACGTGAGCCACGAAGACCCTGAGGTCAAGTTCAACTGGTACGTGGACGGCGTGGAGGTGCATAATGCCAAGACAAAGCCGCGGGAGGAGCAGTACAACAGCACGTACCGTGTGGTCAGCGTCCTCACCGTCCTGCACCAGGACTGGCTGAATGGCAAGGAGTACAAGTGCAAGGTCTCCAACAAAGCCCTCCCAGCCCCCATCGAGAAAACCATCTCCAAAGCCAAAGGGCAGCCCCGAGAACCACAGGTGTACACCCTGCCCCCATCCCGGGATGAGCTGACCAAGAACCAGGTCAGCCTGACCTGCCTGGTCAAAGGCTTCTATCCCAGCGACATCGCCGTGGAGTGGGAGAGCAATGGGCAGCCGGAGAACAACTACAAGACCACGCCTCCCGTGCTGGACTCCGACGGCTCCTTCTTCCTCTACAGCAAGCTCACCGTGGACAAGAGCAGGTGGCAGCAGGGGAACGTCTTCTCATGCTCCGTGATGCATGAGGCTCTGCACAACCACTACACGCAGAAGAGCCTCTCCCTGTCTCCGGGTAAA |
